# Nickel nanoparticles supported by fishbone-derived hydroxyapatite as a bifunctional catalyst for 4-nitrophenol reduction and methanation of carbon oxides

**DOI:** 10.1039/d6ra06935e

**Published:** 2026-09-25

**Authors:** Quang Tri Le, Ngoc Han Lu, Nguyen Khang Vu-Tran, Ai Vi Pham-Nguyen, Thanh Gia-Thien Ho, Ba Long Do, Tri Nguyen, Thi Be Ta Truong

**Affiliations:** a Faculty of Applied Sciences, Ton Duc Thang University Ho Chi Minh City Vietnam lequangtri.st@tdtu.edu.vn lungochan.st@tdtu.edu.vn vutrannguyenkhang.st@tdtu.edu.vn; b Institute of Advanced Technology, Vietnam Academy of Science and Technology No. 1B, TL29 Str., An Phu Dong Ward Ho Chi Minh City Vietnam; c Ho Chi Minh City Open University 97 Vo Van Tan Str. Ho Chi Minh City Vietnam; d Group of Applied Research in Advanced Materials for Sustainable Development, Faculty of Applied Sciences, Ton Duc Thang University Ho Chi Minh City Vietnam truongthibeta@tdtu.edu.vn

## Abstract

Developing low-cost, multifunctional catalysts for aqueous pollutant reduction and gas-phase carbon oxide conversion is important for sustainable environmental technologies. In this study, biogenic HA was extracted from *Pangasius bocourti* fishbone waste and used as a support for NiNPs. A series of *x*Ni/HA catalysts, where *x* = 5, 7.5, 10, and 12.5 wt%, was prepared through wet impregnation, calcination, and hydrogen reduction. The best 10Ni/HA(R) catalyst retained the crystalline HA framework and exhibited an accessible mesoporous and interparticle pore structure. Relatively dispersed metallic NiNPs with an average diameter of 15.20 ± 3.95 nm were observed, while H_2_-TPR indicated the presence of NiO species with different degrees of interaction with the HA support. For aqueous 4-NP reduction, 10Ni/HA(R) achieved complete conversion to 4-AP within 5 minutes at a catalyst dosage of 0.2 g L^−1^ and a 4-NP/NaBH_4_ molar ratio of 1/200. The reaction followed apparent pseudo-first-order kinetics under excess-NaBH_4_ conditions, with *k* = 1.137 min^−1^. During successive 4-NP addition, conversion remained over 93% through ten successive 4-NP additions. The subsequent decrease was mainly associated with progressive NaBH_4_ depletion and reaction-product accumulation in the closed batch system, while post-reaction characterization indicated preservation of the major crystalline phases and a comparable Ni nanoparticle-size range. Under continuous-flow gas-phase methanation conditions at a GHSV of 20 000 h^−1^, the catalyst achieved nearly complete CO conversion at 300 °C and more than 60% CO_2_ conversion at 400 °C. These results demonstrate a promising waste-valorization strategy for producing multifunctional Ni/HA catalysts for aqueous 4-NP reduction and gas-phase carbon oxide conversion.

## Abstract 2

Developing low-cost, multifunctional catalysts for aqueous pollutant reduction and gas-phase carbon oxide conversion is important for sustainable environmental technologies.

## Graphical abstract

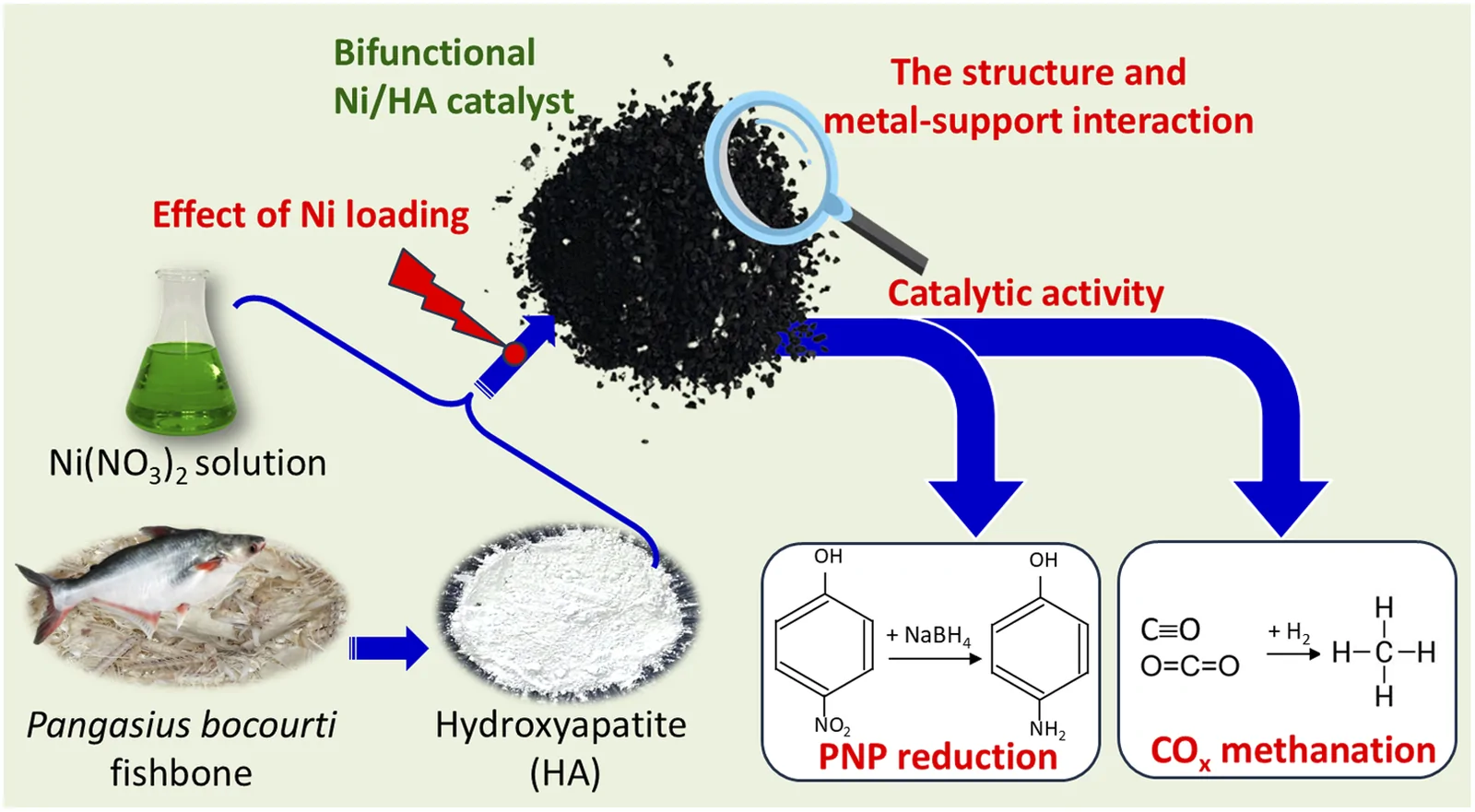

## 1. Introduction

The rapid expansion of the chemical industry to meet societal demands in recent years has led to severe environmental degradation across both aqueous and gaseous phases. Notably, 4-nitrophenol (4-NP) stands out as a highly toxic aqueous nitrophenol derivative, predominantly originating from industrial and agricultural effluents. Owing to its pronounced chemical stability and recalcitrance to biodegradation, 4-NP can persist in the environment, posing severe threats to human health and aquatic ecosystems.^1,2^ Consequently, this compound has been designated as a Priority Pollutant by the United States Environmental Protection Agency (US EPA).^3^ Parallel to water pollution, the deterioration of air quality driven by the continuous emission of carbon oxides is approaching critical levels. Specifically, CO_2_ acts as a primary greenhouse gas accelerating global climate change,^4^ whereas CO is a toxic gas generated from the incomplete combustion of carbonaceous fuels.^5^ Given the high thermodynamic stability of these molecules, their spontaneous degradation under natural conditions is thermodynamically hindered and highly inefficient.^6–8^

Various technologies have been developed for the independent remediation of these pollutants, encompassing advanced oxidation processes,^9^ biological treatments,^10^ and membrane separation^11^ for 4-NP, alongside adsorption processes^12,13^ or electrochemical reduction^14^ for CO_2_ and CO. However, the majority of these conventional techniques are restricted to a single phase or a specific class of pollutants, and frequently suffer from limitations related to high operational costs,^15^ stringent operating conditions,^16^ or suboptimal conversion efficiencies. Photocatalytic processes, for instance, require adequate light irradiation and can therefore become less effective in dark, enclosed, or highly turbid environments. Heterogeneous catalysis provides an alternative route in which waste biomass and biogenic residues can be chemically transformed into value-added products.^17–20^ Among non-noble transition metals, Ni nanoparticles are particularly attractive because of their low cost, abundance, and catalytic activity toward diverse reduction and hydrogenation reactions. In particular, Ni-based catalysts can facilitate the aqueous-phase reduction of 4-NP and the gas-phase methanation of CO_2_/CO,^21,22^ demonstrating their potential for catalytic applications across different reaction environments. However, catalytic performance strongly depends on Ni dispersion, the accessibility of active sites, and interactions with the support, while nanoparticle agglomeration and thermal sintering can lead to activity loss.^23–25^ Immobilizing Ni nanoparticles on a suitable support is therefore an effective strategy for improving their dispersion and stability. From a sustainability perspective, the use of waste-derived supports further provides an opportunity to integrate catalyst development with resource recovery and circular-economy principles.

In Vietnam, the large-scale processing of *Pangasius* generates substantial quantities of fish-bone residues. Owing to their naturally high calcium-phosphate content, these residues provide a readily available biogenic precursor to produce hydroxyapatite (HA). To circumvent the intrinsic limitations of Ni based catalysts, this study utilized HA derived from *Pangasius bocourti* fishbones as the catalytic support.^26^ In addition to valorizing aquaculture by-products in alignment with circular economy principles, HA possesses a hierarchical porous structure, high thermal stability, and an abundance of surface functional groups, which collectively may assist the dispersion and stabilization of Ni species on the support surface.^27,28^ Concurrently, the intrinsic basic sites on the HA framework have been reported to contribute to the adsorption and activation of carbon oxides.^29,30^ Interactions between the active Ni phase and the HA support may therefore influence Ni dispersion, reactant adsorption, and catalytic performance under the investigated reaction conditions.

Grounded in fundamental materials science and the strategic framework of the circular economy, the core objective of this study is to design, prepare, and evaluate a multifunctional Ni/HA heterogeneous catalytic system by valorizing *Pangasius bocourti* fishbone by-products. This material system is investigated in two distinct catalytic applications involving aqueous and gaseous reactants, allowing its catalytic activity and operational behavior to be evaluated under different reaction conditions. Specifically, in the liquid phase, the catalytic performance is evaluated *via* the conversion of the toxic aromatic compound 4-NP into the pharmaceutical precursor 4-aminophenol (4-AP) in the presence of NaBH_4_ as a reducing or hydride-donating agent. Concurrently, in the gaseous phase, the temperature-dependent conversion of CO_2_ and CO under methanation conditions is investigated.

Beyond optimizing the loading of the active Ni phase and evaluating the operational stability of the catalyst during successive 4-NP addition in a closed batch system, this study employs complementary characterization techniques, including HR-TEM, SEM, XRD, BET, H_2_-TPR, and EDS mapping. These characterization techniques were used to provide insight into the structure–activity relationships and possible metal–support interactions. Ultimately, this work presents a promising waste-valorization approach for developing multifunctional biogenic catalyst supports and provides additional insight into the relationships between Ni dispersion, HA-supported structure, and catalytic performance.

## 2. Results and discussion

### 2.1. Characteristics of the Ni/HA catalysts

To elucidate the crystalline phase evolution and the transition behavior of the active Ni phase on the biogenic matrix, powder XRD was performed. Following our previous work which systematically optimized the thermal calcination conditions of the biogenic HA support derived from PB fishbones (650 °C for 3 h),^31^ this study bypasses the support optimization stage and focuses directly on the active phase development on the pre-stabilized HA matrix.

**Fig. 1:**
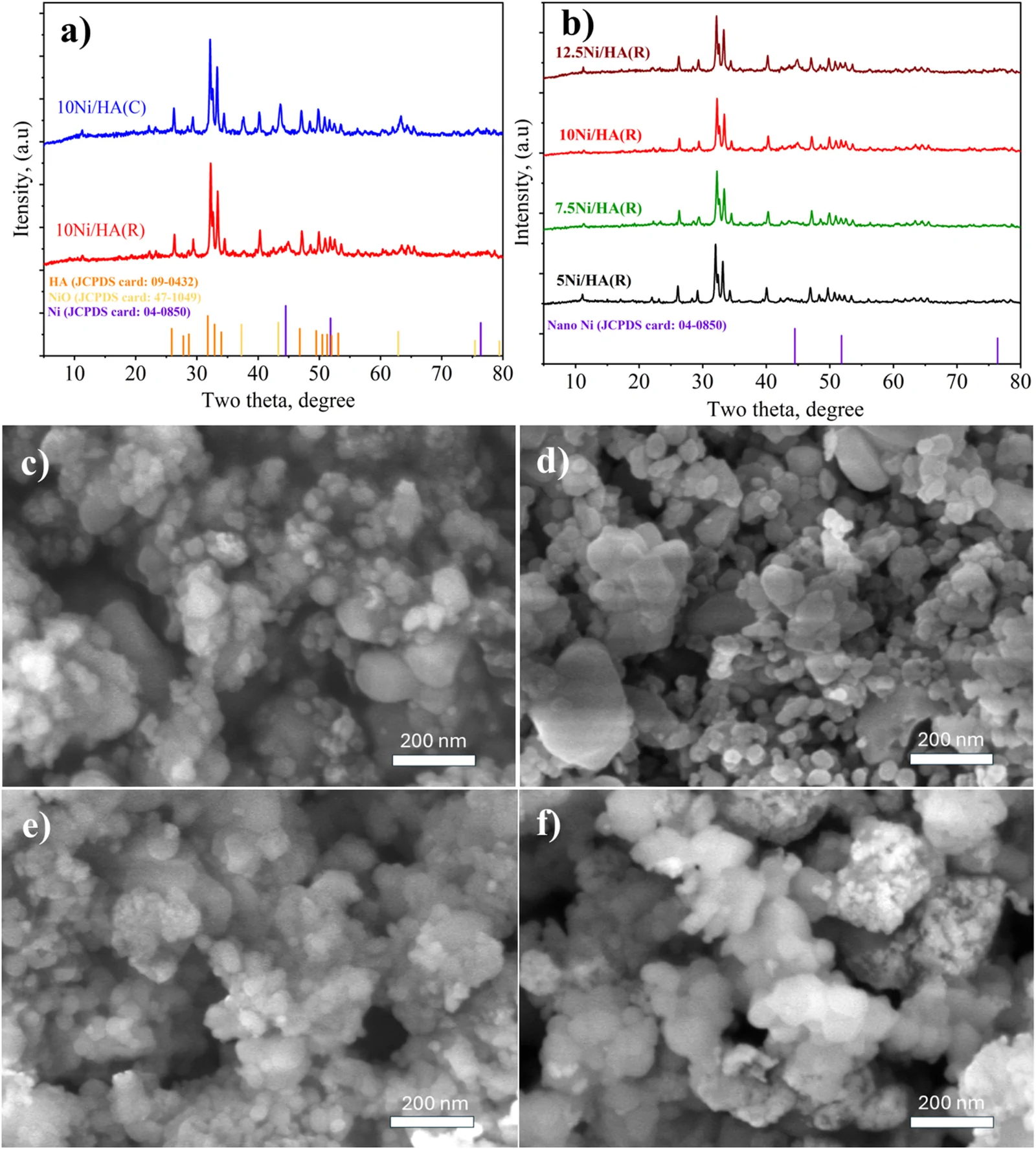
XRD patterns (a and b) and SEM images (c–f) of catalysts.

The comparative XRD profiles of the 10 wt% Ni catalyst after calcination (10Ni/HA(C)) and subsequent hydrogen reduction (10Ni/HA(R)) are shown in Fig. 1a. For both samples, the characteristic diffraction reflections of the biogenic HA carrier (JCPDS card no. 09-0432) remain clearly detectable, with prominent peaks at 2*θ* = 31.8°, 32.9°, 34.0°, 39.8° corresponding to the (211), (300), (202) and (310) Miller indices, respectively.^32^ These observations indicate that the principal crystalline HA phase is retained during impregnation and subsequent thermal processing at 450 °C. However, XRD confirms crystalline-phase preservation rather than the mechanical stability of the support.

In the calcined precursor state (10Ni/HA(C)), three broad diffraction peaks are observed at 2*θ* = 37.2°, 43.3°, and 62.9°, corresponding to the (111), (200), and (220) planes of the cubic NiO phase (JCPDS card no. 47-1049).^28^ This indicates that the calcination of the impregnated Ni(NO_3_)_2_ precursor at 450 °C results in the formation of crystalline NiO species associated with the HA support.^33^ The broadness of these reflections is consistent with relatively small NiO crystallite domains, although their exact spatial distribution and degree of dispersion cannot be established from XRD alone.

Upon undergoing thermal activation under a flowing high-purity hydrogen atmosphere (450 °C for 2 h), a clear change in the crystalline Ni-containing phase is observed. The characteristic diffraction peaks of NiO are no longer detectable within the detection limit of XRD, while a distinct reflection emerges at 2*θ* = 44.5°. This peak corresponds to the (111) crystallographic plane of the FCC Ni^0^ lattice (JCPDS card no. 04-0850).^28^ The appearance of this reflection confirms the formation of crystalline metallic Ni after hydrogen reduction. Although metallic Ni surfaces can contribute to the dissociative adsorption of H_2_, the present XRD data do not establish the Ni(111) plane as the primary active surface or identify the specific atomic configuration of the catalytic sites. Furthermore, the broadening of the Ni(111) peak suggests that the Ni^0^ phase contains relatively small coherent crystallite domains rather than large bulk crystallites. The nanoparticulate nature and particle-size distribution of this phase are more directly supported by the subsequent TEM observations.

To evaluate the impact of metal loading on the dispersion and crystallographic stability of the metallic phase, XRD profiles of catalysts with varying Ni contents (5, 7.5, 10, and 12.5 wt% Ni) were compared, as shown in Fig. 1b. For the catalysts with Ni loadings between 5 wt% and 10 wt%, the diffraction reflections corresponding to Ni^0^ at 2*θ* = 44.5° remain broad, and their intensities generally increase with increasing Ni loading. This trend is consistent with an increasing amount of crystalline metallic Ni while relatively small coherent crystallite domains are maintained up to a loading of 10 wt%. The surface functional groups and defect sites of the biogenic HA may contribute to the anchoring and spatial separation of Ni species, although their specific roles cannot be determined from XRD alone. However, when the Ni loading is further increased to 12.5 wt%, a pronounced change in the Ni(111) reflection is observed. The metallic Ni(111) reflection peak at 2*θ* = 44.5° undergoes a dramatic sharpening accompanied by a substantial surge in diffraction intensity. This peak narrowing is consistent with an increase in the average coherent crystallite size or greater aggregation of the metallic Ni phase. Nevertheless, it should be regarded as qualitative evidence of crystallite growth rather than direct proof of severe thermal sintering.

When the Ni content exceeds the threshold of 10 wt%, the increased local concentration of Ni species may approach or exceed the effective dispersion capacity of the available HA surface sites, promoting the formation of larger metallic crystallites. Such crystallite growth would be expected to reduce the exposed metallic surface area and the number of accessible active sites per unit mass of Ni. Larger surface aggregates may also partially obstruct interparticle voids or pore entrances, although this transport effect cannot be established from XRD alone and should be evaluated together with SEM and N_2_ physisorption results. Taken together, the XRD results suggest that 10 wt% Ni provides the most favorable balance between Ni loading and crystallite dispersion among the investigated samples.

The surface morphology and spatial distribution of the active phase over the biogenic carrier as a function of Ni loading were qualitatively monitored *via* SEM, as illustrated in Fig. 1c–f. Across the catalyst series, the HA-derived framework retains interconnected rod-like and block-like crystallite aggregates together with visible interparticle crevices and voids. These SEM observations describe morphology at the submicrometre scale; the presence and dimensions of mesopores are more appropriately evaluated by N_2_ adsorption–desorption analysis.

For the low-loading catalysts, 5Ni/HA(R) and 7.5Ni/HA(R) in Fig. 1c and d, no extensive formation of large surface aggregates is evident within the observed regions. This morphology is consistent with a relatively dispersed Ni-containing phase at low loading. However, conventional SEM contrast alone cannot conclusively distinguish metallic NiNPs from the HA support or establish their oxidation state and exact location. The observed dispersion may be promoted by interactions between the Ni precursor species and oxygen-containing surface groups on HA, which can limit migration and coalescence during thermal reduction.

At a loading of 10 wt% Ni, Fig. 1e shows a relatively uniform surface morphology without obvious extensive agglomeration within the analyzed field of view. Rather than forming heavy aggregates, the Ni-containing phase appears to remain comparatively dispersed over the HA-derived aggregates. This observation is consistent with favorable metal dispersion at 10 wt% Ni but does not demonstrate a fully homogeneous three-dimensional distribution or selective exposure of Ni(111) facets. The presence of the Ni(111) crystallographic plane is supported by XRD and HRTEM, whereas its surface abundance and specific catalytic role cannot be determined from SEM. Conversely, further elevating the Ni loading to 12.5 wt% in Fig. 1f leads to more pronounced large and block-like surface features, consistent with increased aggregation of the Ni-containing phase. The higher local concentration of Ni precursor species may promote surface migration and particle coalescence during reduction at 450 °C, particularly when the number of available anchoring sites becomes insufficient to maintain the same degree of dispersion. This process results in the formation of larger metallic clusters and localized Ni aggregates on the outer surface of the support. This agglomeration behavior is consistent with the sharpening of the Ni(111) reflection observed in Fig. 1b and would be expected to reduce the accessible metallic surface area. The resulting decrease in accessible active sites may contribute to the lower catalytic rate observed at 12.5 wt% Ni, although direct quantification of metal dispersion would require complementary measurements such as chemisorption or statistically representative TEM analysis.

**Fig. 2:**
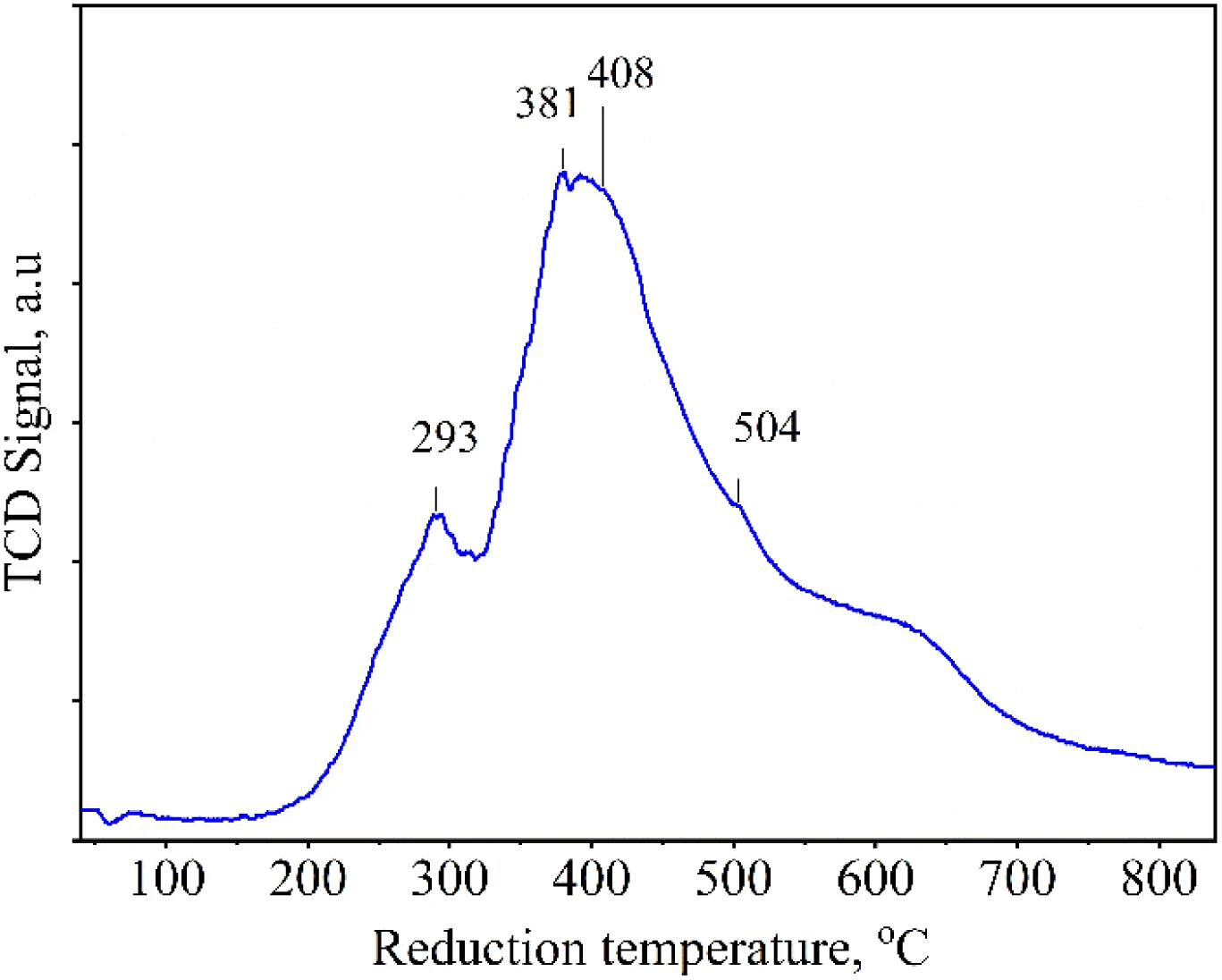
H_2_-TPR profile of 10Ni/HA(C) catalyst.

To examine the redox properties and metal–support interactions of the material, H_2_-TPR was conducted. As shown in Fig. 2, the H_2_-TPR profile of the calcined 10Ni/HA(C) precursor exhibits a broad, asymmetric hydrogen consumption envelope, which exhibits four apparent reduction peaks centered at approximately 293, 381, 408, and 504 °C, suggesting the presence of NiO species with different degrees of interaction with the biogenic carrier.^34^ The first peak, designated as the α peak at 293 °C, is tentatively assigned to the reduction of weakly interacting, highly dispersed surface NiO nanoparticles. The medium-temperature doublet peaks, labelled *β*_1_ (381 °C) and *β*_2_ (408 °C), are tentatively assigned to the reduction of NiO species moderately interacting with the HA support. Lastly, the high-temperature shoulder peak γ centered at 504 °C may be associated with the reduction of strongly interacting NiO species located at the NiO–HA interface, potentially involving Ni–O–Ca or Ni–O–P interfacial environments.^35^ Such interactions may contribute to the anchoring of Ni species on the HA surface and may restrict their migration and coalescence during thermal treatment. However, the exact bonding configuration of these strongly interacting species cannot be conclusively determined from H_2_-TPR alone.

The H_2_-TPR profile supports the selection of 450 °C as a practical catalyst-reduction temperature. The most intense hydrogen-consumption envelope, corresponding mainly to the *β*_1_ and *β*_2_ contributions, is positioned below 450 °C. This observation indicates that reduction at 450 °C is expected to convert most weakly and moderately interacting NiO species into metallic Ni^0^. However, the high-temperature γ contribution extends beyond 450 °C and is centered near 504 °C, suggesting that a fraction of strongly interacting Ni species may not be completely reduced under these conditions. The prolonged isothermal holding at 450 °C for 2 h may promote the gradual reduction of part of these strongly interacting species, although complete reduction cannot be confirmed solely from the present TPR profile.

Using a moderate reduction temperature of 450 °C may also help preserve NiNPs dispersion by limiting excessive thermal migration and particle growth relative to treatment at higher temperatures.

**Fig. 3:**
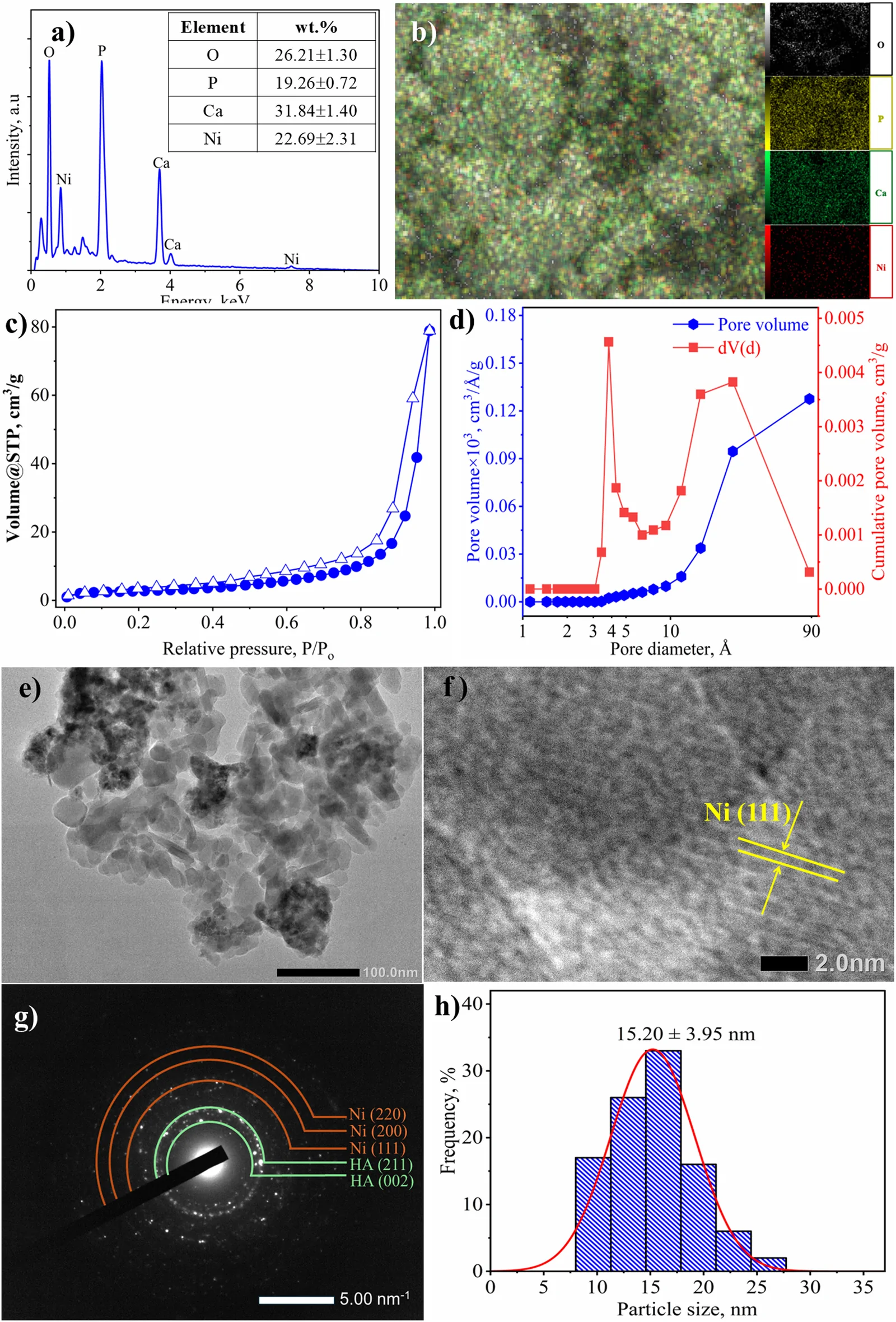
EDS spectrum (a), elemental mapping (b), N_2_ adsorption–desorption isotherms (c), pore size distribution (d); HR-TEM images (e and f), SAED pattern (g), the size distribution of nanoparticles (h) of the fresh 10Ni/HA(R) catalyst.

The surface chemical composition and spatial elemental distribution of 10Ni/HA(R) catalyst were semi-quantitatively evaluated *via* EDS and elemental mapping analysis. As shown in Fig. 3a, the EDS spectrum of the reduced catalyst confirms the presence of Ca, P, O, and Ni within the analyzed region. No additional elemental signals were detected above the detection limit of the technique; however, this observation does not exclude the presence of trace-level impurities. Quantitatively, the analyzed region exhibits an EDS-derived local Ni composition of 22.69 wt%. The significant deviation of this local EDS-derived Ni content from the nominal bulk Ni loading of 10.00 wt% may arise from the localized and semi-quantitative nature of EDS analysis. The interaction volume sampled by EDS typically extends into the near-surface region of the material and depends on the accelerating voltage, sample composition, density, and surface morphology.^36^ Therefore, the measured value should not be interpreted as the actual surface or bulk Ni concentration of the entire catalyst. During wet impregnation and subsequent thermal reduction at 450 °C, the Ni precursor species nucleate and form nanoparticles primarily on the external HA facets, within larger interparticle crevices, and near pore entrances. The relatively high local Ni signal may therefore indicate enrichment of Ni-containing particles within the specific region selected for EDS analysis. In addition, the presence of NiNPs over the HA substrate may influence the relative X-ray intensities recorded for Ca, P, and O through local coverage, matrix effects, and differences in the interaction volume. Nevertheless, the extent of these effects cannot be quantified from the present EDS data alone. Correspondingly, the EDS-derived local Ca/P atomic ratio of 1.28, calculated from 23.09 at% Ca and 18.07 at% P, differs from the theoretical stoichiometric value of 1.67 for pure HA.^37^ Because EDS measurements are sensitive to the selected analysis area, sample roughness, particle orientation, local heterogeneity, and matrix corrections, this local Ca/P ratio should not be interpreted as the bulk stoichiometry of the HA support. The discrepancy does not necessarily indicate bulk structural degradation, as the characteristic HA reflections remain detectable in the XRD patterns. However, XRD primarily reflects the bulk crystalline phases and cannot independently exclude local surface compositional variations. The deviation in the local Ca/P ratio may therefore reflect a combination of regional compositional heterogeneity, partial coverage of the HA substrate by Ni-containing particles, surface roughness, and limitations inherent to semi-quantitative EDS analysis. Accordingly, the present EDS results support the presence of locally concentrated Ni-containing regions but do not provide direct evidence of a uniform surface-enrichment mechanism or establish a quantitative relationship between Ni enrichment and catalytic activity.

As illustrated by the elemental mapping in Fig. 3b, Ca, P, O, and Ni are spatially distributed throughout the analyzed region. The Ni elemental map indicates a relatively dispersed distribution without obvious micron-scale segregation or large localized Ni-rich domains within the field of view. This observation is consistent with the TEM evidence of dispersed NiNPs; however, EDS mapping alone cannot determine the oxidation state of Ni, confirm three-dimensional homogeneity throughout the entire catalyst, or prove that the HA support suppresses thermal clustering.

To evaluate the actual textural characteristics and pore accessibility of the stabilized channel network following Ni deposition, N_2_ adsorption–desorption physisorption analysis was performed at 77.3 K (Fig. 3c). This physisorption study provides deeper quantitative insights into the specific surface area, pore volume, and pore size distribution of 10Ni/HA(R) catalyst. The catalyst exhibits a characteristic Type IV isotherm according to the IUPAC classification, which is accompanied by a distinct H3-type hysteresis loop situated in the relative pressure (*P*/*P*_0_) range of 0.45 to 0.99 (Fig. 3c). This specific thermodynamic isotherm profile is indicative of a well-developed mesoporous architecture. From a textural perspective, rather than a crystallographic one, the H3-type hysteresis loop is closely associated with slit-shaped capillary pores or wedge-like channel configurations formed by the loose aggregation of plate-like and rod-like HA crystallites, which is consistent with, rather than conclusively proven by, the structured mineral aggregates observed in our FESEM micrographs. In the low relative pressure region (*P*/*P*_0_ lower than 0.05), the adsorption curve remains nearly flat without a pronounced initial uptake, suggesting that microporosity makes only a limited contribution to the total accessible surface area. Nevertheless, the sub-nanometer feature observed in the BJH pore-size distribution should be interpreted cautiously because the conventional BJH method is not sufficiently reliable for quantitative pore-size determination in the micropore region below approximately 2 nm. Quantitatively, the multi-point BET calculation yielded an *S*_BET_ of 9.7 m^2^ g^−1^, with a total *V*_PORE_ of 0.127 cm^3^ g^−1^. The BJH desorption analysis yielded a higher apparent cumulative pore area of 24.4 m^2^ g^−1^. However, the BET specific surface area and the BJH cumulative pore area are derived from different models and relative-pressure regions and should therefore not be considered directly equivalent measurements. In an H3-type pore network, capillary evaporation, pore-blocking effects, and the idealized pore-geometry assumptions of the BJH model may increase the apparent cumulative contribution of smaller pores. Accordingly, the BET value was used as the principal specific surface area, whereas the BJH analysis was primarily used to describe the approximate distribution of accessible pore widths.

While these values appear relatively modest compared to conventional high surface area synthetic carriers (such as SBA-15 or activated carbons),^38,39^ they must be scientifically interpreted through both the intrinsic nature of the biomineralized framework and the metal loading configuration. First, unlike synthetic HA, which is frequently prepared by mild wet-chemical precipitation and may exhibit relatively low crystallinity, the biogenic HA extracted from PB fishbones underwent high-temperature calcination at 650 °C for 3 h. This thermal treatment induced grain crystallization, minimized lattice defect density, and thermal coalescence of the primary grains. This necessary recrystallization stabilizes the inorganic matrix against structural collapse but naturally compromises the specific surface area, leading to a highly crystalline, dense, and naturally low surface area skeleton. Second, Ni deposition may partially cover or obstruct accessible pore entrances. During wet impregnation and subsequent thermal reduction at 450 °C, Ni precursor species nucleate to form metallic NiNPs. Given their average diameter of 15.20 ± 3.95 nm, the metallic NiNPs are expected to be located mainly on external HA facets, within larger interparticle crevices, and near the entrances of smaller pores, where they may partially restrict pore accessibility. Because the average NiNPs diameter is substantially larger than the predominant BJH pore-width range, the NiNPs cannot be geometrically confined inside the 1.5–3.0 nm pores. Instead, these small pores represent accessible textural features of the HA-based composite, whereas the NiNPs are predominantly located on external surfaces, larger interparticle voids, and pore-mouth regions.

Such deposition may partially cover or obstruct accessible pore entrances. This partial obstruction may reduce the accessibility of nitrogen molecules to some internal pore regions and may contribute to the measured BET surface area and total pore volume. The ability of the biogenic HA support to maintain NiNPs may therefore be related to its surface functional groups, rough hierarchical morphology, and spatially separated nucleation regions, rather than to the direct geometrical confinement of entire 15 nm NiNPs within 1.5–3.0 nm pores. These surface features may help limit the lateral migration and coalescence of Ni species during thermal reduction. The relatively low BET surface area did not preclude the observed catalytic activity. In the present system, catalytic performance may depend more directly on the accessibility and local dispersion of the metallic Ni phase than on the total BET surface area of the support alone. The combined TEM and elemental-mapping results are consistent with a relatively dispersed Ni-containing phase within the analyzed regions.

Furthermore, as illustrated by the BJH pore-size distribution in Fig. 3d, the pore-width distribution exhibits an apparent bimodal profile, with a sharp apparent sub-nanometer feature at approximately 0.4 nm and a broader distribution spanning approximately 1.5–3.0 nm. The 1.5–3.0 nm interval crosses the IUPAC boundary between micropores and mesopores, with the dominant mesoporous contribution centered at approximately 2.5–3.0 nm. The accessible interparticle pore network may provide transport pathways that facilitate the diffusion of liquid-phase reactants, including 4-NP, and the circulation of gaseous reactants, including CO and CO_2_, while supporting the desorption of reaction products. However, these pores should not be described as cavities containing the entire NiNPs or as channels hosting active sites deep within the HA matrix.^40^

The nanoscale morphology, internal microstructure, and crystallographic lattice properties of 10Ni/HA(R) catalyst were directly visualized and thoroughly analyzed *via* TEM, HR-TEM, and SAED, as illustrated in Fig. 5e–h. As shown in the TEM image (Fig. 3e), the active metallic NiNPs exhibit a predominantly near spherical morphology and are interspersed across the biogenic HA matrix, without any severe local clustering or broad particle agglomeration. A closer inspection reveals that the NiNPs are closely associated with interfacial regions of the fishbone-derived HA matrix. From a materials science perspective, these biogenic boundary steps may provide surface functional groups and possible anchoring sites, which may interact with Ni^2+^ precursor species during wet impregnation and contribute to the stabilization of dispersed Ni species during subsequent hydrogen reduction.^41^

To resolve the atomic arrangement and surface characteristics of the active metal phase, HRTEM analysis was conducted (Fig. 3f). The highly magnified image reveals distinct, ordered periodic lattice fringes on the spherical metallic nanoparticle. Crucially, the resolved lattice spacing yields an interplanar *d*-spacing of 0.203 nm, which is assigned to the (111) crystallographic plane of the FCC Ni^0^ phase. This direct atomic-scale observation confirms the presence of the Ni(111) crystallographic plane in the active nanoparticles, which may provide a favorable surface coordination environment for catalytic activation.

While the lattice fringes of the underlying biogenic HA support are not resolved in this specific localized HR-TEM projection due to contrast and orientation limits under the high energy electron beam, the physical association of the NiNPs with the HA support matrix can still be observed. Furthermore, the structural integrity and high crystallinity of the hexagonal HA framework are supported by bulk XRD patterns and the subsequent SAED rings, which clearly exhibit the characteristic reflections of HA.

The SAED pattern (Fig. 3g) further corroborates the highly crystalline nature of both phases, while successfully resolving the multiple crystallographic planes of the system. The SAED image exhibits a series of bright, sharp, concentric Debye–Scherrer diffraction rings. The inner concentric rings are indexed to the (002) and (211) reflections of the hexagonal HA carrier, confirming that its crystal structure remains intact after thermal reduction.^42^ Extending outward, the subsequent distinct rings are indexed to the (111), (200), (220), and (311) crystallographic planes of the crystalline metallic Ni^0^ phase.^43^ The coexistence of these sharp, well-resolved diffraction rings, without prominent diffuse amorphous halos in the analyzed region, supports the preservation of crystallinity in both phases after thermal calcination and hydrogen activation.

To quantitatively evaluate the size control of the active phase, a statistical particle size distribution histogram was constructed by measuring about 100 individual nanoparticles (Fig. 3h). The catalyst exhibits a relatively narrow, approximately unimodal particle-size distribution, yielding an average metallic NiNPs size of 15.20 ± 3.95 nm. This relatively narrow distribution is consistent with the ability of the biogenic HA support to maintain NiNPs under the applied preparation conditions.^44^ Its hierarchical morphology, natural crevice-like layout, and abundant surface anchoring sites provide spatially separated nucleation domains that hinder the lateral migration and coalescence of active Ni species, resulting in an average particle size of approximately 15 nm, with most measured particles remaining in the nanoscale range.^34,45^

To further evaluate the uniqueness and efficacy of the developed biogenic support, the average metallic Ni particle size of 10Ni/HA(R) catalyst was compared with various recently reported HA-supported Ni catalysts in the literature, as summarized in Table S1. When compared with catalysts prepared *via* the same wet-impregnation method, the developed biogenic system exhibits a promising and efficient control over the active phase grain size.^45^ Specifically, 10Ni/HA(R) catalyst with a high Ni loading of 10 wt% achieves a well dispersed active phase with an average particle size stabilized at 15.20 ± 3.95 nm. This represents a reasonable and eco-efficient balance between maintaining a nanoscale active phase dispersion and process simplicity, leveraging a facile, promoter-free, and low-cost wet-impregnation route over a sustainable seafood-waste-derived support.

In contrast, Nguyen *et al.* reported an average Ni crystallite size of 24.5–25.8 nm for a similar 10 wt% Ni catalyst supported on salmon bone-derived HA prepared using a broadly similar wet-impregnation route.^28^ Furthermore, even with the addition of a ceria modifier (the 10Ni-6Ce/HA catalyst), their particle size remained relatively large at 25.8 nm.^28^ Compared with these reported values, the smaller average Ni size measured for the present catalyst suggests that differences in support morphology, surface chemistry, and preparation conditions may have favored Ni dispersion. The oxygen-containing surface groups and hierarchical morphology of the PB fishbone-derived HA may provide favorable sites for Ni deposition and may help limit particle migration and coalescence during reduction at 450 °C, without the addition of a foreign promoter.

The comparatively narrow particle-size distribution of the present catalyst can also be considered alongside those reported for catalysts prepared using other impregnation approaches. Alonso *et al.* reported that a 10 wt% Ni/HA catalyst prepared by incipient wetness impregnation exhibited an average Ni size of 27.0 nm and a broad size distribution of ±18.0 nm, consistent with greater particle growth or aggregation in that particular system.^46^ The broad distribution reported for this particular IWI-prepared catalyst may reflect differences in precursor distribution, support properties, and thermal-treatment conditions. In contrast, the present wet-impregnation route yielded a comparatively narrow particle-size distribution of 15.20 ± 3.95 nm within the analyzed TEM regions. This result may reflect the combined effects of the impregnation conditions and the surface characteristics of the biogenic HA support.

Notably, our catalyst maintains competitive performance despite its higher metal loading. Gong *et al.* synthesized a 3Ni/HA catalyst with only 3.0 wt% Ni loading, which yielded an average particle size of 15.3 nm.^47^ Lower metal loadings can generally reduce the probability of particle growth and aggregation, although the resulting metal dispersion also depends strongly on the support properties and preparation conditions.^34,48^ However, our 10Ni/HA(R) catalyst, despite carrying more than three times the active metal content (10.0 wt%), still maintains a slightly smaller average particle size (15.20 nm). This result suggests that the PB fishbone-derived HA support and the applied preparation route may help maintain NiNPs even at a Ni loading of 10 wt%. While sub-10 nm Ni particles can be achieved through complex or modified routes, such as using highly sensitive and expensive organometallic precursors (10Ni@HA, 4.4 nm)^46^ or multi-step foreign ion doping (Ni/Sr-HA, 9.2 nm),^49^ these methods involve significant economic and environmental drawbacks that hinder large-scale industrial application.

Consequently, the biogenic 10Ni/HA(R) catalyst developed in this work offers a promising and eco-efficient alternative for environmental and energy applications. It demonstrates a reasonable balance between maintaining a nanoscale active phase dispersion and process simplicity, leveraging a facile, promoter-free, and low-cost wet-impregnation route over a sustainable seafood waste-derived support.

### 2.2. Kinetic analysis and catalytic performance in 4-nitrophenol reduction

Building upon the comprehensive structural, textural, and redox insights obtained through our XRD, BET, H_2_-TPR, EDS Mapping, SEM and HR-TEM/SAED analyses, the practical applicability of Ni/HA catalysts was systematically evaluated. Specifically, the liquid-phase catalytic reduction of 4-NP to 4-AP in the presence of NaBH_4_ was employed as a model reaction^50^ to directly correlate the catalyst's physicochemical properties with their surface reaction kinetics.^51^

Due to the massive stoichiometric excess of the NaBH_4_ reductant relative to the organic pollutant, the concentration of the active hydride donor species remains practically constant throughout the reaction course. Consequently, the overall reaction rate depends solely on the temporal concentration of the limiting 4-NP reactant, thereby validating the application of the apparent pseudo-first-order kinetic model (1):1
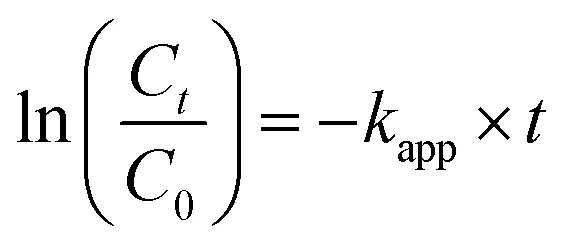
where *C*_0_ and *C*_*t*_ represent the concentrations of 4-NP at the initial state and at reaction time *t* (min), respectively, and *k*_app_ denotes the apparent first-order rate constant (min^−1^).

**Fig. 4:**
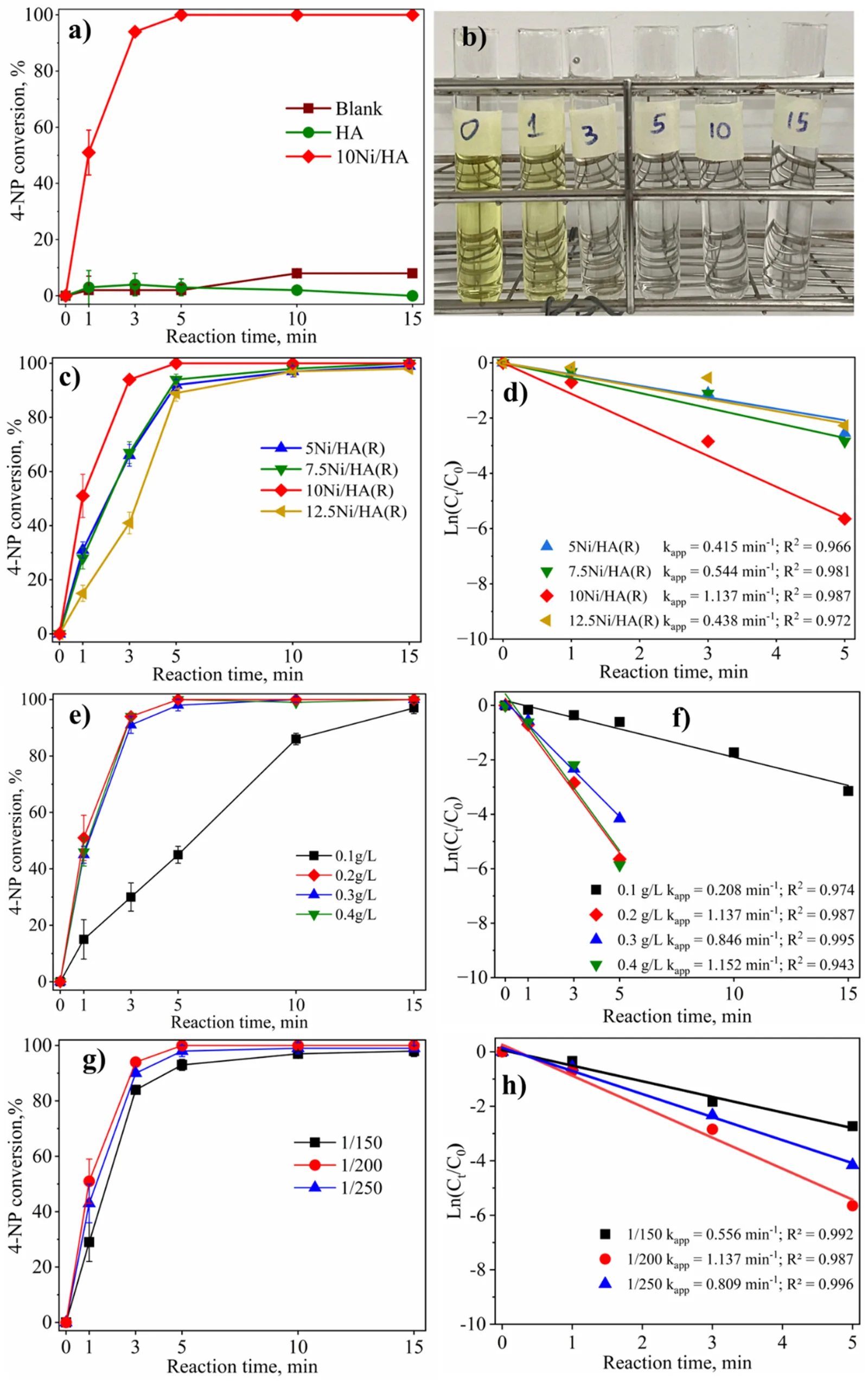
The 4-NP reduction process over the Ni/HA catalyst: conversion efficiency comparison over the blank control (without catalyst), bare biogenic HA support, and 10Ni/HA(R) catalyst (a); color of reaction solutions over time using 10Ni/HA(R) catalyst (b); effect of active Ni loading (c and d); effect of the 10Ni/HA catalyst dosage (e and f); and effect of the 4-NP/NaBH_4_ molar ratio (g and h).

As illustrated in Fig. 4a, control experiments conducted in the absence of a catalyst (blank) or using the bare biogenic HA support yielded negligible 4-NP conversion (below 9% after 15 min), indicating that the uncatalyzed reduction proceeds slowly under the applied conditions. This limited conversion may be associated with the kinetic barrier to interfacial electron or hydride transfer between BH_4_^−^ and 4-nitrophenolate species in the aqueous medium. Conversely, the introduction of Ni/HA catalysts accelerated the reaction kinetics as visually evidenced by the decolorization of the solution over time in Fig. 4b, indicating that the Ni-containing surface provides an alternative catalytic pathway with a lower apparent kinetic barrier. The catalytic activity exhibits a strong, non-monotonic dependence on the active phase loading (Fig. 4c). As calculated from the linear plots in Fig. 4d, the apparent rate constant increases progressively in the order: 5Ni/HA(R) (*k*_app_ = 0.415 min^−1^) < 7.5Ni/HA(R) (*k*_app_ = 0.544 min^−1^) < 10Ni/HA(R) (*k*_app_ = 1.137 min^−1^).

This steady kinetic enhancement is consistent with the microstructural characterization results. From a crystallographic perspective, XRD analysis (Fig. 1b) confirms the formation of crystalline metallic Ni, while HR-TEM observations (Fig. 3f) reveal NiNPs with an average size of 15.20 ± 3.95 nm and lattice fringes assigned to the Ni(111) plane. These results confirm the presence of nanoscale metallic Ni domains but do not establish Ni(111) as the uniquely dominant or intrinsically most active surface. The relatively dispersed Ni-containing phase may increase the number of accessible metallic sites available for reactant adsorption and may facilitate interfacial electron or hydride transfer from the BH_4_^−^ donor to the 4-NP acceptor.^52^

Based on control experiments and related NaBH_4_-assisted dark reduction studies, the catalytic role of the Ni-containing surface can be interpreted through a surface-mediated donor–acceptor pathway. In the alkaline reaction medium, 4-NP is predominantly present as 4-nitrophenolate, whereas BH_4_^−^ acts as the reducing donor. Previous studies on AgMoOS@AC, Mn/O-Bi_2_S_3_, and CuSnOS/AC have proposed that adsorption of BH_4_^−^ derived reducing species and pollutant molecules on catalytic surfaces facilitates interfacial electron and hydrogen-species transfer during pollutant reduction.^53–55^ By analogy, the accessible metallic Ni domains in the present Ni/HA system may act as interfacial relay sites that facilitate the transfer of reducing equivalents toward adsorbed 4-nitrophenolate, followed by the formation and desorption of 4-AP. However, the mixed-valence redox couples and vacancy-mediated charge-transfer pathways reported for these oxysulfide catalysts have not been directly established for the present Ni/HA system and are therefore not specifically assigned here.

To evaluate the influence of catalyst dosage on the performance of 10Ni/HA(R), the catalyst concentration was varied from 0.1 to 0.4 g L^−1^. As shown in Fig. 4e and f, increasing the dosage from 0.1 to 0.2 g L^−1^ increased *k*_app_ from 0.208 to 1.137 min^−1^, which is consistent with the greater number of accessible catalytic sites present in the reaction system. Further increases in catalyst dosage did not produce a consistent improvement in the apparent reaction rate: *k*_app_ decreased to 0.846 min^−1^ at 0.3 g L^−1^ and increased to 1.152 min^−1^ at 0.4 g L^−1^. Although 0.4 g L^−1^ yielded the numerically highest *k*_app_, this value was only approximately 1.3% higher than that obtained at 0.2 g L^−1^, despite the twofold increase in catalyst dosage. Therefore, 0.2 g L^−1^ was selected as a practical catalyst dosage that provided a near-maximum apparent reaction rate while limiting additional catalyst consumption. The non-monotonic behavior above 0.2 g L^−1^ may reflect changes in suspension density, mixing, mass transfer, or reducing-species availability; however, the individual contributions of these factors were not quantified in the present study.

Finally, the influence of the reductant concentration (Fig. 4g and h) highlights the competitive adsorption dynamics at the catalyst interface. The highest rate constant (*k*_app_ = 1.137 min^−1^) was achieved at a 4-NP/NaBH_4_ molar ratio of 1/200. Elevating the ratio to 1/250, however, resulted in a lower *k*_app_ value of 0.809 min^−1^. This decline may be associated with: (i) the excessive generation of gaseous H_2_ bubbles that physically cover the catalyst facets, reducing the active solid–liquid contact area, and (ii) the competitive adsorption of surplus BH_4_^−^ ions, which crowd the metallic surface and block the co-adsorption of 4-nitrophenolate molecules. These explanations remain proposed contributions because gas evolution, surface coverage, and adsorption competition were not directly quantified in the present study.

In summary, the comprehensive kinetic profiling confirms that 10Ni/HA(R) catalyst, utilizing a sustainable and eco-efficient biogenic fishbone support, represents a promising and highly viable candidate for aqueous environmental remediation. Its representative apparent rate constant at the selected catalyst dosage, *k*_app_ = 1.137 min^−1^, obtained under the selected operating conditions of 0.2 g L^−1^ catalyst dosage and a 4-NP/NaBH_4_ molar ratio of 1/200, is consistent with the relatively dispersed Ni phase and accessible external and interparticle morphology.

To provide a broader assessment of the catalytic performance of the biogenic 10Ni/HA(R) catalyst, its activity was benchmarked against both HA-supported catalysts and recently reported heterogeneous catalysts for NaBH_4_-assisted 4-NP reduction. Because the reported systems differ in catalyst composition, catalyst dosage, reductant amount, reaction volume, and other experimental conditions, the comparison in Table 1 is intended as an indicative performance benchmark rather than a direct measure of intrinsic catalytic activity. The catalyst dosage, reaction time, and apparent pseudo-first-order rate constants were therefore considered together when evaluating the reported catalytic systems.

**Table 1:** Catalytic performance comparison of various heterogeneous catalysts for the reduction of 4-NP

Catalyst	Active phase (wt%)	Support material	*D*_cat_, g L^−1^	*t*, min^−1^	*k*_app_, min^−1^	Ref.
Ag/PDA-HAP	Ag (47.86)	Polydopamine – coated HAP	0.06	5	0.438	58
Ag/HA	Ag (1.87)	Chemical HA	0.8	5	0.680	57
0.7% Cu/HA	Cu (0.67)	Chemical 1D HA	0.1	6	0.710	56
Sr/S-W_18_O_49_	Sr/S-co-doped W_18_O_49_	Unsupported	0.03	6	0.360	59
Fe/O-Bi_2_S_3_-3	Fe/O-co-doped Bi_2_S_3_	Unsupported	0.05	6	0.620	23
10Ni/HA	Ni (10.0)	PB fishbone HA	0.2	5	1.137	This work

As illustrated in Table 1, biogenic 10Ni/HA(R) catalyst exhibits a competitive apparent rate constant of 1.137 min^−1^, achieving complete 4-NP conversion within 5 minutes under a practical catalyst dosage of 0.20 g L^−1^. This performance represents a favorable kinetic enhancement compared to conventional non-noble transition metal systems. Specifically, the reported *k*_app_ of the present catalyst is approximately 1.6 times that of the chemical 1D HA supported copper catalyst (0.7% Cu/HA, *k*_app_ = 0.710 min^−1^), despite the copper based system requiring a longer reaction time of 6 minutes.^56^ Under the respective reported conditions, this comparison indicates that the present Ni/HA system exhibits competitive apparent reduction kinetics relative to the Cu/HA catalyst. However, differences in active-metal loading, catalyst dosage, and reaction conditions preclude a direct comparison of the intrinsic catalytic activities of Ni and Cu.

When evaluated against noble metal decorated catalysts on similar HA based supports, our non-noble system exhibits highly competitive performance, demonstrating the unique structural advantages of the biogenic carrier. For instance, the Ag/HA catalyst reported by Hoang *et al.* (2020) utilized chemical HA to support precious silver (1.87 wt% Ag).^57^ To achieve complete 4-NP reduction in 5 minutes, this chemical HA supported system required a high catalyst dosage of 0.80 g L^−1^ (four times higher than our system) and exhibited a *k*_app_ value of 0.680 min^−1^, compared with 1.137 min^−1^ for the present 10Ni/HA(R) catalyst.

This faster kinetic rate becomes apparent when compared to the high loading Ag/PDA-HAP (HAP5) system synthesized by Das *et al.* (2018). While HAP5 achieved reaction completion in 5 minutes with a low catalyst dosage (0.06 g L^−1^), it required large active phase loading of 47.86 wt% Ag and yielded a rate constant of only 0.438 min^−1^.^58^ By contrast, our 10Ni/HA system achieves a rate constant nearly three times higher using entirely low-cost, earth-abundant Ni on a sustainable fish-bone support, avoiding expensive noble metals and complex polydopamine surface modifications.

Beyond HA-supported systems, several recently developed defect-engineered catalysts have also demonstrated rapid NaBH_4_-assisted reduction of 4-NP. For example, Sr/S-W_18_O_49_ achieved complete reduction of 20 mg L^−1^ 4-NP within 6 min using a catalyst dosage of 0.03 g L^−1^, with a reported *k*_app_ of 0.36 min^−1^.^59^ Similarly, Fe/O-Bi_2_S_3_-3 completely reduced 100 mL of 20 mg L^−1^ 4-NP within 6 min using a catalyst dosage of 0.05 g L^−1^ and exhibited a *k*_app_ of 0.62 min^−1^.^23^ Under the selected conditions of the present study, 10Ni/HA(R) achieved complete conversion within 5 min with a representative *k*_app_ of 1.137 min^−1^ at a catalyst dosage of 0.20 g L^−1^. Although the present system exhibits a higher apparent rate constant under the respective reported conditions, the substantially different catalyst dosages and reaction conditions preclude a direct comparison of intrinsic catalytic activity based solely on *k*_app_. Accordingly, these results indicate that the fishbone-derived Ni/HA system exhibits competitive 4-NP reduction kinetics while retaining the advantages of a promoter-free preparation route and a waste-derived HA support.

The favorable performance is consistent with the microstructural and textural properties discussed in the preceding sections. First, TEM and HR-TEM analyses in Fig. 3e and f show relatively dispersed NiNPs with an average size of 15.20 ± 3.95 nm and lattice fringes assigned to the Ni(111) plane. The hierarchical surface morphology, larger interparticle crevices, and oxygen-containing anchoring sites of the HA support may provide spatially separated nucleation regions that limit excessive migration and coalescence of Ni species during reduction. Second, N_2_ adsorption–desorption analysis in Fig. 3c and d indicates a Type IV isotherm with an H3-type hysteresis loop and a narrow mesopore distribution, with a dominant mesoporous contribution near 2.5–3.0 nm. The NiNPs are not located inside these small pores but are mainly associated with external surfaces, larger interparticle voids, and pore entrances. The accessible pore network may support reactant transport toward these externally accessible Ni-containing sites, although unrestricted intraparticle diffusion cannot be claimed from the present data. Finally, the H_2_-TPR profile in Fig. 2 indicates NiO species with different degrees of interaction with the HA support. These interactions may contribute to Ni anchoring and the preservation of nanoscale dispersion during catalyst activation. However, H_2_-TPR does not directly prove that such interactions prevent leaching during liquid-phase operation or establish the intrinsic turnover rate of the catalyst.

### 2.3. Successive 4-NP addition test and post-reaction characterization

**Fig. 5:**
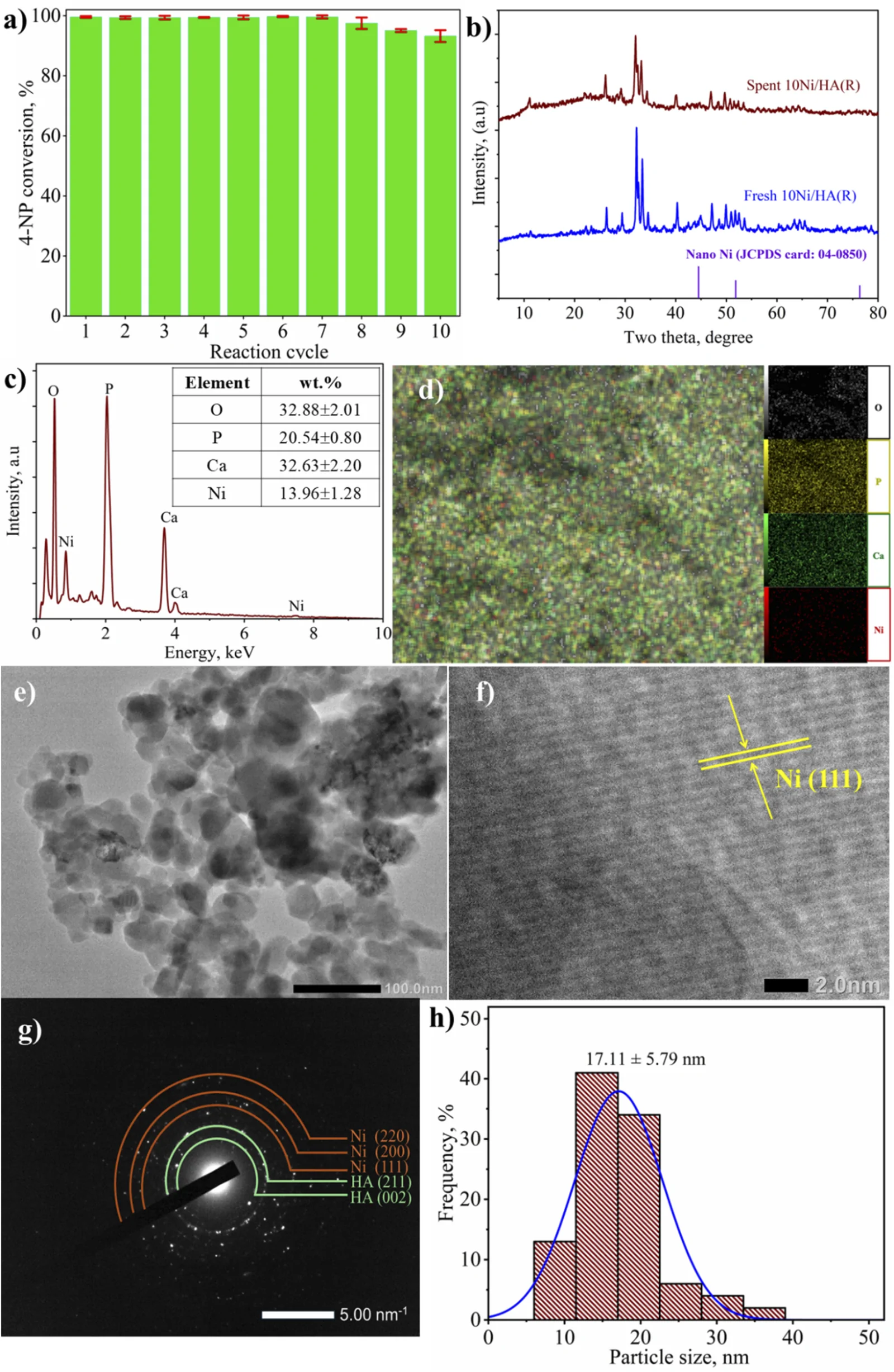
Operational stability of 10Ni/HA(R) over 10 successive 4-NP additions (a); XRD comparison of fresh 10Ni/HA(R) and spent 10Ni/HA(S) (b); EDS spectrum (c), elemental mapping (d), images of HR-TEM (e and f), SAED pattern (g), and the particle size distribution (h) of the spent 10Ni/HA(S) catalyst.

The long-term stability of a heterogeneous catalyst under prolonged reaction conditions is a critical criterion governing its economic viability and environmental sustainability for practical applications. Therefore, the durability of the 10Ni/HA(R) catalyst was evaluated through ten successive 4-NP additions in a closed batch system, without recovering the catalyst or replenishing the initially added NaBH_4_ reducing agent. Accordingly, this experiment is interpreted as a successive-substrate-addition operational stability test rather than a conventional catalyst recovery-and-reuse test. As illustrated by the conversion trends across ten consecutive PNP addition cycles in Fig. 5a, the 10Ni/HA catalyst exhibited virtually no loss of stability during the first seven successive additions of 4-NP; specifically, it maintained a 4-NP conversion efficiency exceeding 99% throughout these initial runs. However, the apparent conversion rate declined by 2% per cycle from the 8th to the 10th addition, with the PNP conversion efficiency dropping slightly to 93% by the 10th cycle. Because neither fresh NaBH_4_ nor a renewed reaction medium was supplied during the experiment, this decline cannot be attributed exclusively to intrinsic catalyst deactivation. Instead, it may reflect the combined effects of changes in the catalyst and the progressive evolution of the closed reaction medium.

To examine whether the decline in conversion was associated with major structural changes, the spent catalyst recovered after the tenth addition, denoted 10Ni/HA(S), was subjected to comprehensive post-reaction characterization. From a crystallographic perspective, the XRD pattern of the spent catalyst (Fig. 5b) shows that the characteristic reflections of the hexagonal HA support and the FCC Ni^0^ phase (at 2*θ* ≈ 44.5°) remain detectable after ten successive 4-NP additions in the aqueous reaction medium. No additional crystalline reflections attributable to NiO, Ni(OH)_2_, or other secondary crystalline phases were observed within the detection limit of XRD, indicating that no major crystalline phase transformation or structural collapse of the HA support occurred under the applied reaction conditions. However, small quantities of poorly crystalline or amorphous surface species cannot be excluded solely on the basis of XRD. Concurrently, HR-TEM analysis of the spent sample (Fig. 5e and f) shows that the NiNPs remain associated with the biogenic HA support. Lattice fringes assigned to the Ni(111) plane remain observable in the analyzed regions. The measured average NiNPs size changed from 15.20 ± 3.95 nm in the fresh catalyst to 17.11 ± 5.79 nm in the sample spent (Fig. 5h). Considering the overlap between the two particle-size distributions, this result does not indicate extensive particle growth or severe agglomeration during the room-temperature liquid-phase reaction. However, without statistical significance testing, the observed difference should not be regarded as definitive evidence of particle coalescence. Furthermore, the SAED concentric rings (Fig. 5g) support the preservation of the major crystalline HA and Ni phases.

To evaluate the chemical preservation of the active phase, EDS and mapping analyses were performed on the spent 10Ni/HA(S) catalyst. EDS analysis (Fig. 5c) records a local Ni concentration of 13.96 wt% in the spent catalyst, compared with 22.69 wt% in the fresh material. Because EDS provides local and semi-quantitative elemental information, the lower Ni signal may reflect partial Ni loss, differences between the analyzed regions, or surface coverage by adsorbed reaction-derived species. Therefore, the EDS results alone should not be regarded as direct quantitative evidence of Ni leaching. ICP-OES analysis showed Ni contents of 11.60 wt% and 11.01 wt% for the fresh and spent catalysts, respectively, which are reasonably consistent with the theoretical Ni loading of 10 wt%. These bulk ICP-OES results also support that the higher local Ni content observed by EDS is associated with the localized nature of EDS analysis and the non-uniform surface distribution of Ni species. The relatively small difference between the fresh and spent catalysts further suggests that most of the Ni was retained in the catalyst after the reaction. Nevertheless, the elemental mapping images in Fig. 5d indicate that the remaining Ni is distributed across the observed HA support region without severe localized aggregation. This observation is consistent with the preservation of a relatively dispersed Ni-containing phase but does not independently prove the nature or strength of the metal–support interactions.

Based on the post-reaction characterization and the closed-batch experimental design, the pronounced decrease in conversion during the tenth addition is proposed to arise from a combination of the following factors. First, because fresh NaBH_4_ was not supplied after each 4-NP addition, the initially added reducing agent was progressively consumed during the target reduction reaction and through competing hydrolysis. The resulting decrease in the availability of active borohydride species likely limited the reaction during the tenth addition. Second, repeated addition of 4-NP without replacing the reaction solution resulted in the progressive accumulation of 4-AP and other reaction-derived species. Their adsorption on the catalyst surface may have reduced the accessibility of Ni sites to newly added 4-NP and BH_4_^−^.

While the closed-batch conditions eventually restricted the overall reaction performance during the tenth addition because of reductant depletion, product accumulation, and possible changes in the accessible Ni phase, the performance maintained during the first nine successive additions demonstrates favorable operational tolerance of the biogenic 10Ni/HA(R) catalyst under the applied conditions. Nevertheless, the present test does not independently establish conventional catalyst reusability because the catalyst was not recovered and exposed to completely renewed 4-NP and NaBH_4_ solutions after each run. The observed preservation of the major crystalline phases and nanoparticle-size range supports further evaluation of this low-cost biogenic catalyst for environmental remediation. Future recovery-and-reuse experiments using fresh reaction solutions after each run would be required to distinguish intrinsic catalyst deactivation from reductant depletion, product accumulation, and possible metal loss. Continuous-flow operation with a controlled reactant and reductant supply may also reduce the closed-batch limitations observed in the present experiment.

### 2.4. Catalytic activity in CO_2_ and CO conversion

To evaluate the multifunctional capability of the developed biogenic catalyst system, the conversion of CO and CO_2_ under methanation conditions was investigated over 10Ni/HA(R) catalyst. Thermochemically, the activation of these carbon oxide molecules presents distinct thermodynamic and kinetic challenges. While CO_2_ is a highly stable, linear symmetric molecule with a high double bond energy (C=O bond strength of approximately 805 kJ mol^−1^),^60^ CO possesses a strong triple bond configuration (1072 kJ mol^−1^),^61^ making low-temperature conversion dependent on the availability of active metallic sites and potentially influenced by interactions with the support surface.

**Fig. 6:**
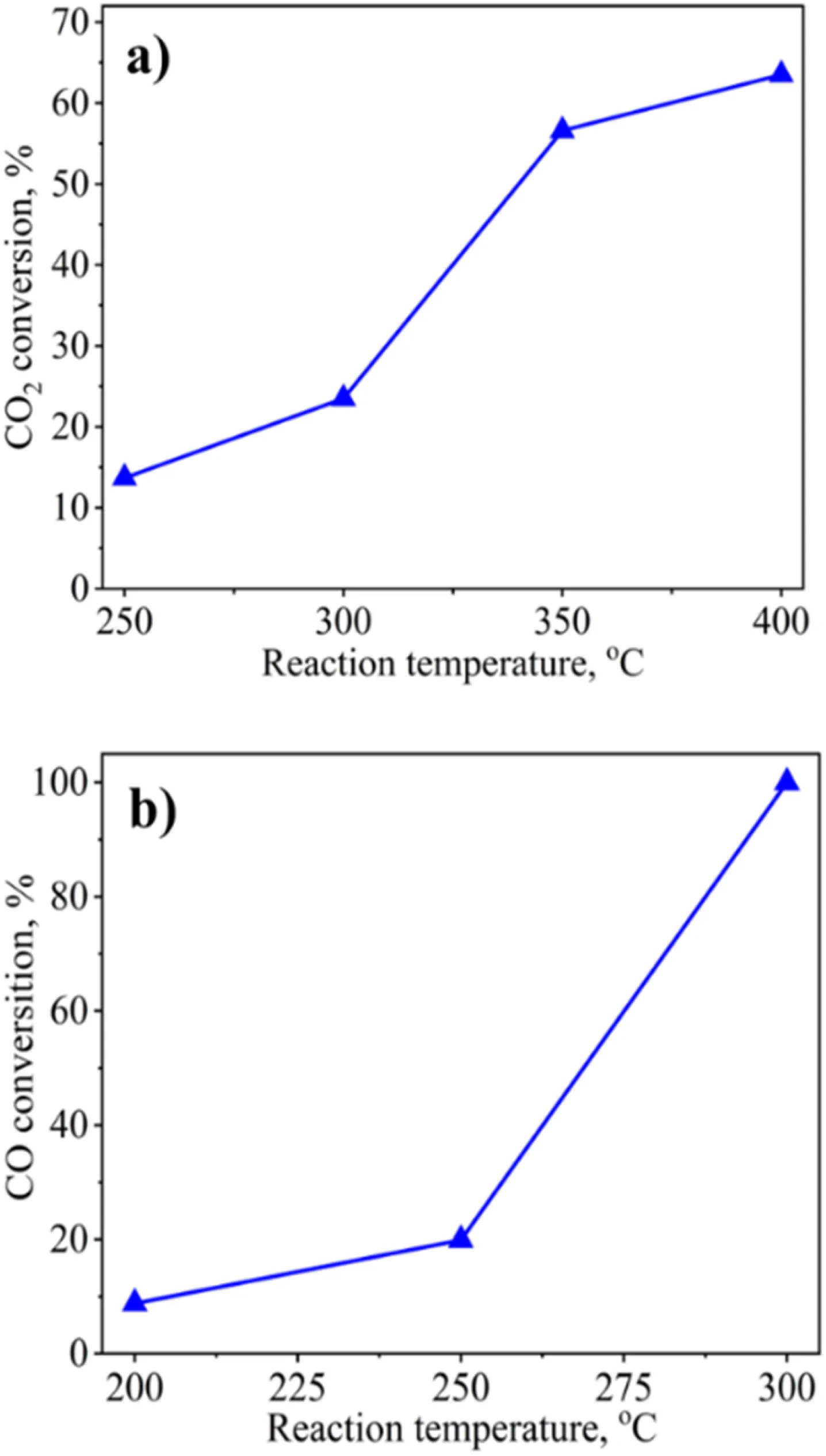
Catalytic activity of 10Ni/HA(R) in the conversion of CO_2_ (a) and CO (b) at GHSV = 20 000 h^−1^.

As illustrated by the conversion profiles in Fig. 6a, the catalytic conversion of CO_2_ over the 10Ni/HA catalyst exhibits a strong, positive temperature dependence within the surveyed range of 200–400 °C. At a GHSV of 20 000 h^−1^, the conversion of CO_2_ increases rapidly with temperature, surpassing 60% conversion at 400 °C. This kinetic behavior is consistent with the structural and chemical characteristics of our biogenic catalytic system. Specifically, at the crystallographic level, the crystalline metallic NiNPs with an average size of 15.20 ± 3.95 nm exhibit lattice fringes assigned to the Ni(111) plane with a *d*-spacing of 0.203 nm. The presence of metallic Ni surfaces may facilitate the dissociative adsorption of H_2_ and the formation of reactive hydrogen species; however, the present characterization does not establish selective exposure of Ni(111) or identify this plane as the uniquely dominant active surface.^62,63^ Concurrently, the biogenic HA carrier derived from PB fishbone waste exhibits an accessible mesoporous and interparticle pore structure, with a dominant mesoporous contribution near 2.5–3.0 nm. Oxygen-containing groups and potentially basic surface environments on HA may contribute to CO_2_ adsorption and activation.^64^

Hydrogen transfer or spillover from adjacent Ni sites may also contribute to the subsequent hydrogenation of adsorbed carbon-containing species, but this pathway remains a proposed mechanism.^65^ Furthermore, the accessible pore and interparticle-void structure may facilitate the transport of gaseous reactants and the removal of reaction products, including water vapor. However, the present data do not establish the absence of intraparticle mass-transfer limitations or confirm resistance to water-induced deactivation or reoxidation of metallic Ni during operation.^45^

Notably, under gas-phase methanation conditions, the 10Ni/HA(R) catalyst exhibited appreciable low-temperature CO conversion at a GHSV of 20 000 h^−1^, as shown in Fig. 6b. CO conversion was 8.8% at 200 °C, increased to 20.0% at 250 °C, and reached nearly complete conversion at 300 °C. Below 250 °C, the low conversion is consistent with kinetic limitations associated with the adsorption, activation, and hydrogenation of CO-derived surface species.^66^ However, the present conversion data alone are insufficient to identify CO dissociation as the rate-determining step or to establish a distinct transition between kinetic regimes. The rapid increase in CO conversion indicates that the apparent kinetic limitations become less pronounced at 300 °C; however, comparison with thermodynamic equilibrium requires calculations based on the actual feed composition, pressure, and temperature.^67^

The favorable low-temperature CO conversion under methanation conditions may be related to the morphological and surface chemical properties of the biogenic catalyst. First, the interaction of chemisorbed CO with basic or defect sites on the HA surface may assist the adsorption and activation of CO-derived surface species. However, direct weakening or cleavage of the C≡O bond on the HA surface was not demonstrated in the present study. Second, the dispersed metallic NiNPs, with an average size of 15.20 ± 3.95 nm, exhibit lattice fringes assigned to the Ni(111) plane. The metallic Ni surfaces may provide sites for the dissociative adsorption of H_2_. Hydrogen spillover from Ni to adjacent support sites may contribute to the hydrogenation of adsorbed intermediates, but this pathway still remains a proposed mechanism. The combined roles of accessible Ni sites and the HA surface may therefore facilitate CO adsorption, H_2_ activation, and subsequent hydrogenation steps. Nevertheless, the present results do not demonstrate that the catalyst bypasses a specific direct CO-cleavage pathway. Under the applied conditions, the catalyst achieved nearly complete CO conversion at 300 °C under a spatial velocity of 20 000 h^−1^.^45^

To place the catalytic performance of the biogenic 10Ni/HA(R) catalyst within the context of previously reported systems, its catalytic metrics were compared with those of other Ni/HA catalysts reported in the recent literature. As summarized in Table 2, this evaluation includes unpromoted and promoter-assisted HA-supported catalysts, providing an indicative comparison under broadly comparable reaction temperatures and gas hourly space velocities.

**Table 2:** Comparative evaluation of Ni/HA catalysts for CO_2_ methanation

Catalyst	wt% Ni	Support	GHSV, h^−1^	*T*, °C	*X*_CO_2__, %	Ref.
10Ni/HA	10	Biogenic salmon bone HA	20 000	400	48.2	28
10Ni-6Ce/HA	10	CeO_2_ – promoted salmon HA	20 000	400	58.5	28
10Ni/HA	10	Chemical HA	12 000	400	51.0	46
10Ni/HA	10	PB fishbone HA	20 000	400	60.0	This work

In a conventional study reported by Nguyen *et al.*, the 10Ni/HA catalyst synthesized from salmon bone-derived HA *via* a standard wet-impregnation method yielded relatively large Ni crystallites ranging from 24.5 to 25.8 nm. This restricted metal dispersion may have reduced the accessible metallic surface area, and the addition of 6 wt% CeO_2_ was subsequently investigated to improve the methanation performance of the resulting 10Ni-6Ce/HA catalyst.^28^ Similarly, Alonso *et al.* reported thermal sintering of metallic Ni on conventional chemical HA supports, resulting in a crystallite size of approximately 27.0 nm that may have reduced the accessible active-site density.^46^

Conversely, as indicated by HR-TEM observations, the natural porous structure and surface defects of the biogenic HA carrier derived from PB fishbones may provide potential anchoring sites for Ni species. These surface features may contribute to the dispersion and stabilization of the active Ni phase, yielding an average metallic NiNPs size of 15.20 ± 3.95 nm with a relatively narrow size distribution. The unpromoted 10Ni/HA(R) catalyst in this work achieved a CO_2_ conversion of over 60% at 400 °C and a GHSV of 20 000 h^−1^. Under the respective reported conditions, this catalytic performance compares favorably with several reported Ni-based catalysts supported on synthetic metal oxides, including the Ni/CeO_2_ system reported by Ye *et al.*,^68^ highlighting the potential environmental and economic benefits of valorizing Vietnamese aquaculture waste.

Indeed, the low temperature activity of unpromoted 10Ni/HA(R) catalyst is further highlighted in CO conversion reaction, where nearly 100% conversion was achieved at a relatively low temperature of 300 °C under the high space velocity of 20 000 h^−1^. For comparison, conventional transition metal catalysts or support-modified systems reported in the literature often require much lower space velocities or the addition of precious metal promoters to achieve complete CO removal at this temperature range. The biogenic PB fishbone-derived HA support may help stabilize dispersed NiNPs exhibiting lattice fringes assigned to Ni(111) (*d* spacing of 0.203 nm), thereby promoting CO adsorption, H_2_ activation, and subsequent hydrogenation without the use of expensive chemical promoters.

The catalytic activity observed in 4-NP reduction and carbon oxide conversion is consistent with the presence of accessible Ni sites and possible interactions between the Ni-containing phase and the biogenic HA support. The hydroxyl groups, basic surface environments, and hierarchical morphology of HA may contribute to reactant adsorption and the stabilization of Ni species during catalyst preparation and operation. However, the specific roles of these surface sites, as well as resistance to carbon deposition and long-term thermal deactivation, require further investigation. Overall, the waste-derived 10Ni/HA(R) catalyst shows promising potential for further development in aqueous pollutant reduction and continuous gas-phase carbon oxide conversion.

## 3. Experimental

### 3.1. Materials

Ca Bien Ho Seafood Import-Export Joint Stock Company, located in An Giang Province, Vietnam, supplied *Pangasius bocourti* (PB) fish bones as the primary precursor for hydroxyapatite (HA) synthesis. Distilled water and high-purity gases, including hydrogen (H_2_, 99.999%), nitrogen (N_2_, 99.999%), carbon dioxide (CO_2_, 99.999%), carbon monoxide (CO, 99.999%) were sourced locally in Vietnam. Nickel(ii) nitrate hexahydrate (Ni(NO_3_)_2_·6H_2_O, 98.0 wt%), sodium borohydride (NaBH_4_, 99.0 wt%), 4-nitrophenol (4-NP, 99.0 wt%) were purchased from commercial suppliers in China. All chemicals and reagents utilized in this study were of analytical grade and were used as received without any further purification.

### 3.2. Preparation of HA from PB fishbones

The preparation protocol of HA from PB fishbones was adapted from our previous study^31^ and shown in Fig. S1a. Upon collection, the PB fishbones were thoroughly washed with water, then cleaned fishbones were subsequently heated under pressurized aqueous conditions for 1 hour to remove the remaining soft tissues and adhering organic matter. Subsequently, the bone samples were dried at 110 °C for 12 hours to eliminate moisture. The dried material was then subjected to calcination in air at 650 °C for 3 hours, based on the optimized conditions established in our previous study, to substantially remove residual organic components and obtain the crystalline HA phase. Following the calcination process, the samples were allowed to cool naturally to room temperature and finely ground to obtain the powdered HA material, which was subsequently utilized for further experimental procedures.

### 3.3. Preparation of Ni/HA catalysts

The synthesis procedure of the catalyst is illustrated in Fig. S1b, where an amount of *x* g of the finely ground HA powder was dispersed into V mL of an aqueous Ni(NO_3_)_2_·6H_2_O solution and left to impregnate for 2 hours, facilitating the uniform adsorption of Ni^2+^ precursors onto the support surface. Subsequently, the suspension was subjected to a sequential drying process at 80 °C and 120 °C, for 2 hours at each isothermal stage, to evaporate the solvent and stabilize the precursor distribution throughout the material. The dried precursor was then calcined at 450 °C for 2 hours to thermally decompose the nitrate species, resulting in the formation of a well-dispersed NiO phase anchored on the HA framework. Ultimately, the material was subjected to a reduction treatment under a continuous H_2_ stream at 450 °C for 2 hours to convert Ni^2+^ into metallic Ni^0^, thereby generating highly dispersed metallic active sites over the support surface.

### 3.4. Catalytic evaluation

#### 3.4.1 Liquid-phase 4-NP reduction

The catalytic activity of the Ni/HA catalysts was evaluated using the NaBH_4_-mediated reduction of 4-NP as a model reaction. Two control experiments were conducted under otherwise identical conditions: a blank experiment without catalyst and a support-control experiment containing bare HA without Ni. These controls were used to assess the extent of uncatalyzed 4-NP reduction and adsorption by the HA support, respectively, thereby distinguishing these contributions from the activity of the Ni-containing catalyst. Three experimental variables were investigated: the nominal Ni loading, the catalyst dosage, and the initial 4-NP/NaBH_4_ molar ratio. The percentage conversion of 4-NP at reaction time *t* was calculated using eqn (2):2
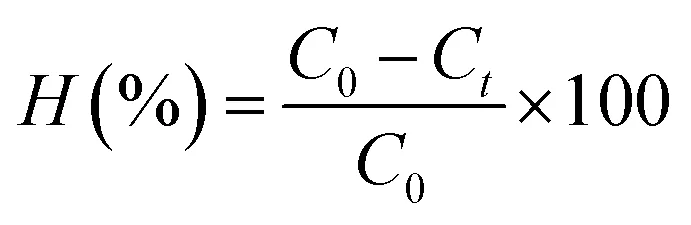
where *C*_0_ and *C*_*t*_ denote the initial 4-NP concentration and the concentration at reaction time *t*, respectively, and *H* represents the percentage conversion of 4-NP. The calculated conversion values were used to compare the catalytic performance of different catalyst compositions and reaction conditions. Experiments were performed in duplicate, and the reported values represent the means of the two measurements.

#### 3.4.2 Methanation of carbon oxides

The catalytic performance for the methanation of CO_2_ and CO was evaluated in a continuous-flow fixed-bed quartz reactor at atmospheric pressure. In this study the reaction temperature was systematically varied from 200 to 400 °C) at a constant gas hourly space velocity (GHSV) of 20 000 h^−1^. The catalytic efficiency was quantified by determining the conversion of CO (*X*_CO_) and CO_2_ (*X*_CO_2__) calculated according to eqn (3) and (4), respectively:3
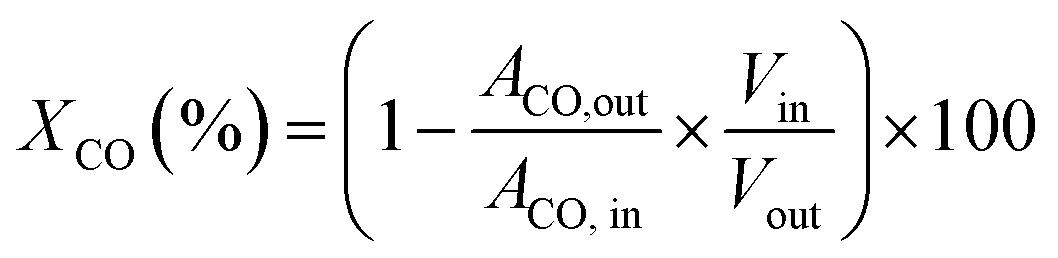
4
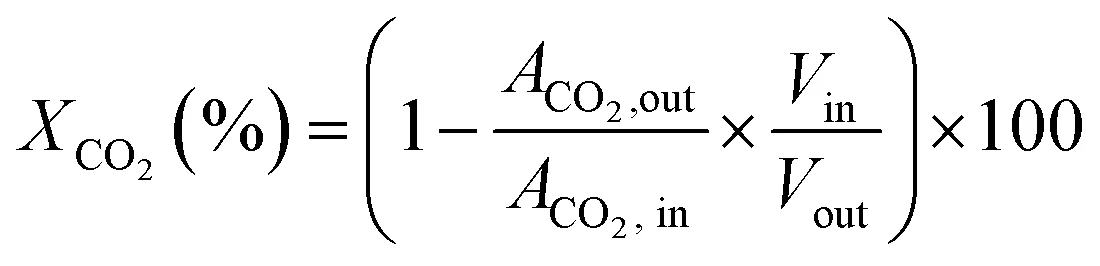
where *A*_CO,in_ and *A*_CO,out_ denote the GC peak areas of the corresponding carbon oxide in the feed and product streams, and *V*_in_ and *V*_out_ represent the gas flow rates measured by a soap-film flowmeter at the reactor inlet and outlet. The product gas stream was sampled manually and analyzed quantitatively using a gas chromatograph (Agilent Technologies 6890 Plus) equipped with a Thermal Conductivity Detector (TCD). All catalytic tests were performed in duplicate to ensure data reproducibility, and the results are presented as average values.

### 3.5. Characterization

The structural, morphological, textural, and chemical properties of the Ni/HA catalysts were examined using complementary characterization techniques. The progress of the aqueous 4-NP reduction reaction was monitored by ultraviolet-visible spectroscopy (UV-vis, Shimadzu UV-1800) through the time-dependent changes in the characteristic absorption bands. Product composition analysis and the evaluation of the independent methanation efficiencies of CO_2_ and CO were conducted utilizing gas chromatography (GC, Agilent Technologies 6890 Plus). The crystalline phase composition and degree of crystallinity were determined *via* powder X-ray diffraction (XRD, Bruker D2-PHASER) using CuKα radiation (*λ* = 1.5406 Å). The specific surface area and textural properties were evaluated *via* N_2_ adsorption–desorption isotherms at 77.3 K using the Brunauer–Emmett–Teller (BET) technique. Surface morphology and elemental composition were investigated using field-emission scanning electron microscopy coupled with energy-dispersive X-ray spectroscopy (FE-SEM/EDS, JEOL JST-IT 200). Meanwhile, the microstructural features, NiNPs size, and crystallographic lattice properties were analyzed utilizing high-resolution transmission electron microscopy combined with selected area electron diffraction (HR-TEM/SAED, JEOL JEM 2100). The reducibility of the metal phases and the interactions between the active species and the support were investigated by hydrogen temperature-programmed reduction (H_2_-TPR).

## 4. Conclusions

In summary, a multifunctional and promoter-free 10Ni/HA(R) heterogeneous catalyst was successfully prepared using biogenic HA derived from PB fishbone waste. The catalyst retained the crystalline HA framework and exhibited an accessible mesoporous and interparticle pore structure, together with relatively dispersed metallic NiNPs with an average diameter of 15.20 ± 3.95 nm. XRD and HRTEM confirmed the formation of crystalline metallic Ni and lattice fringes assigned to the Ni(111) plane, while H_2_-TPR indicated the presence of NiO species with different degrees of interaction with the HA support. For aqueous 4-NP reduction, the catalyst achieved complete conversion to 4-AP within 5 minutes at the selected catalyst dosage of 0.2 g L^−1^ and a 4-NP/NaBH_4_ molar ratio of 1/200. Under these selected conditions, the reaction followed apparent pseudo-first-order kinetics with *k*_app_ = 1.137 min^−1^. This dosage provided a near-maximum reaction rate while limiting additional catalyst consumption. During the successive-addition test, the conversion efficiency remained at a minimum of 93% over ten successive 4-NP additions. The subsequent decrease was interpreted as being associated with progressive NaBH_4_ depletion and reaction-product accumulation in the closed batch system. Post-reaction characterization showed preservation of the major crystalline phases and a comparable NiNPs-size range, although the lower local Ni signal observed by EDS may reflect partial Ni loss, regional variability, or surface coverage. Under continuous-flow gas-phase methanation conditions at a GHSV of 20 000 h^−1^, the catalyst achieved nearly complete CO conversion at 300 °C and more than 60% CO_2_ conversion at 400 °C. The observed performance is consistent with the presence of accessible metallic Ni sites, external HA surfaces, interparticle transport pathways, and possible interfacial interactions that may contribute to Ni stabilization. Overall, this study demonstrates a promising waste-valorization strategy for converting fishbone-derived HA into a multifunctional catalyst support for aqueous 4-NP reduction and gas-phase carbon oxide conversion.

## Author contributions

Quang Tri Le, Ngoc Han Lu, Nguyen Khang Vu-Tran: methodology, resources, validation, investigation, formal analysis, writing – original draft. Ai Vi Pham-Nguyen, Thanh Gia-Thien Ho, Ba Long Do: methodology, investigation, validation, formal analysis. Tri Nguyen, Thi Be Ta Truong: supervision, conceptualisation, methodology, writing – original draft, writing – review & editing.

## Conflicts of interest

The authors declare that there is no conflict of interest regarding the publication of this paper.

## Supplementary Material

RA-OLF-D6RA06935E-s001

## Data Availability

The data used to support the findings of this study are included within the article. Supplementary information (SI) is available. See DOI: https://doi.org/10.1039/d6ra06935e.
