# Supplementary material for: Nickel nanoparticles supported by fishbone-derived hydroxyapatite as a bifunctional catalyst for 4-nitrophenol reduction and methanation of carbon oxides
Source: RSC Adv. 2026 Sep 25;16(45):57499–516. doi: 10.1039/d6ra06935e (PMC13613653; doi:10.1039/d6ra06935e)
Supplement: Supplementary file 1 [file RA-016-D6RA06935E-s001.pdf]

Supplementary Information (SI) for RSC Advances.
This journal is © The Royal Society of Chemistry 2026

Supplementary material

Fishbone-derived hydroxyapatite supported nickel nanoparticles as a bifunctional catalyst for 4-nitrophenol reduction and methanation of carbon oxides

Quang Tri Le[a,+], Ngoc Han Lu[a,+], Nguyen Khang Vu-Tran[a,+], Ai Vi Pham-Nguyen[b], Thanh Gia-Thien Ho[b], Ba Long Do[b], Tri Nguyen[b,c], Thi Be Ta Truong[d,*]

[a]*Faculty of Applied Sciences, Ton Duc Thang University, Ho Chi Minh City, Vietnam (email: lequangtri.st@tdtu.edu.vn; lungochan.st@tdtu.edu.vn; vutrannguyenkhang.st@tdtu.edu.vn)*

[b]*Institute of Advanced Technology, Vietnam Academy of Science and Technology, No.1B, TL29 Str., An Phu Dong ward, Ho Chi Minh City, Vietnam*

[c]*Ho Chi Minh City Open University, 97 Vo Van Tan Str., Ho Chi Minh City, Vietnam*

[d]*Group of Applied Research in Advanced Materials for Sustainable Development, Faculty of Applied Sciences, Ton Duc Thang University, Ho Chi Minh City, Vietnam*

**Corresponding author: truongthibeta@tdtu.edu.vn*

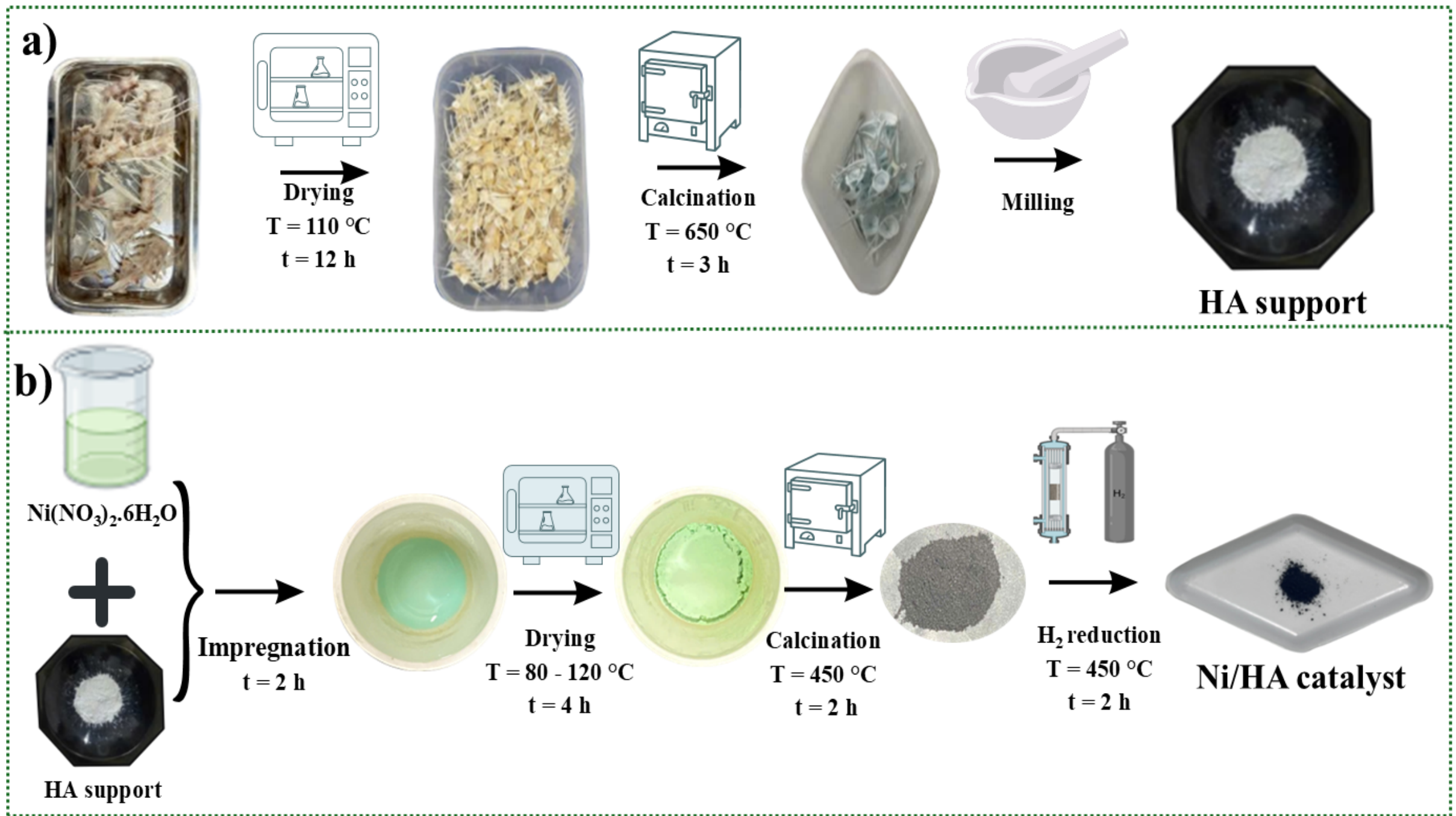

Fig. S1 Pretreatment and preparation process of HA support from PB fishbones (a) and the synthesis of the Ni/HA catalyst (b)

Tab. S1 Physicochemical property comparison of various supported Ni-based catalysts.

Catalyst	Preparation method	Nanoparticle size, nm	Refs.
10Ni/HA	Wet-impregnation	15.20±3.95	This work
10Ni@HA	Organometallic precursor	4.4	[1]
10Ni/HA_IWI	Incipient wetness impregnation	27.0±18.0	[1]
Ni/Sr-HA	Wet-impregnation (Strontium modified)	9.2	[2]
3Ni/HA	Wet-impregnation	15.3	[3]
10Ni-6Ce/HA	Wet-impregnation (Ceria modified)	25.8	[4]
10Ni/HA	Wet-impregnation	24.5–25.8	[4]

References:

1. A. P. Alonso, A. Serrano-Maldonado, J.-B. Ledeuil, L. Madec, D. P. Minh, D. Pla and M. Gómez, ACS Sustain. Resour. Manag., 2024, 1, 451-461.
2. N. Berroug, M. A. Gutiérrez-Ortiz, J. R. González-Velasco and Z. Boukha, Journal of Industrial and Engineering Chemistry, 2025, 149, 764-777.
3. B. Gong, T. Su, X. Xie, H. Ji and Z. Qin, Ind. Eng. Chem. Res., 2023, 62, 12935-12948.
4. T. T. V. Nguyen, N. Phung Anh, T. G.-T. Ho, T. T. P. Pham, P. H. D. Nguyen, B. L. Do, H. K. P. Huynh and T. Nguyen, ACS Omega, 2022, 7, 36623-36633.
